# Functionalized Core/Shell Gold-Palladium Bimetallic Nanoparticles in Transferrin-Targeted Dual-Drug Delivery in a Cervical Cancer Cell Model

**DOI:** 10.3390/ph19010074

**Published:** 2025-12-30

**Authors:** Lorenzo Lance David, Moganavelli Singh

**Affiliations:** Nano-Gene and Drug Delivery Group, Discipline of Biochemistry, University of KwaZulu-Natal, Private Bag X54001, Durban 4000, South Africa; lorenzolance@gmail.com

**Keywords:** bimetallic nanoparticles, gold, palladium, drug delivery, 5-fluorouracil, doxorubicin, transferrin, chitosan, encapsulation

## Abstract

**Background/Objectives:** Research on noble metal nanoparticles (NPs) has increased over the past three decades, with advancements in synthesis techniques refining their physicochemical characteristics, including size, shape, and surface chemistry. Bimetallic NPs (BNPs) offer synergistic properties contributed by both metals. Gold (Au) and palladium (Pd) NPs possess low toxicity, high biocompatibility and loading, ease of synthesis and surface modification. Doxorubicin (DOX) and 5-fluorouracil (5-FU) are potent chemotherapeutic drugs but are rapidly metabolised in the body, producing severe side effects, limiting their use. Hence, innovative strategies to mitigate this is needed. **Methods:** In this study, AuPd NPs in a core-shell formation were chemically synthesized. The AuPd NPs were conjugated to 5-FU and DOX-encapsulated CS complexes and decorated with the targeting moiety transferrin (Tf). **Results:** Transmission electron microscopy and nanoparticle tracking analysis confirmed that the BNPs were spherical, with an average size of 73.4 nm. Functionalized BNPs were able to encapsulate more than 70% of 5-FU and DOX, resulting in a controlled drug release profile at pH 4.2. Cytotoxicity levels in human cancer cells, HeLa (cervical carcinoma) and MCF-7 (breast adenocarcinoma), as well as in non-cancer HEK293 (embryonic kidney) cells, revealed that the Tf-targeted nanocomplexes were HeLa cell-specific, with no significant cytotoxicity in the HEK293 cells. Tf-mediated cellular uptake was confirmed by receptor competition studies in the HeLa cells. Apoptosis and oxidative stress analysis confirmed cell death by apoptosis, consistent with the action of 5-FU and DOX. **Conclusions:** This study highlighted the potential of this BNP-nanocomplex as a suitable vehicle for drug delivery.

## 1. Introduction

Cancer development can be attributed to a complex network of genetic, physiological, and environmental factors. The global 2020 cancer statistics reflected over 19 million new cancer cases, with approximately 10 million deaths, with new cases expected to reach over 28 million by 2040 [1]. These predictions underscore the need to enhance classical therapeutic approaches, which have historically been suboptimal [2]. Cervical cancer is the fourth leading cancer in women and is a significant global concern, with 2020 statistics reporting 342,000 mortalities and 604,000 new cancer cases [3]. Although various factors could influence cancer development, the human papillomavirus is present in the majority of cervical cancer cases [4]. Despite numerous advances in both primary and secondary prevention, cervical cancer has been shown to progress quickly towards metastasis, often within the first year of primary treatment [5]. In these cases, surgical resection is recommended; depending on the level of disease progression, treatment could include radiation therapy. However, chemotherapy is the only viable treatment option for most patients due to the disease’s aggressive nature [4,5].

Historically, conventional chemotherapeutic approaches have been associated with severe side effects, often exacerbating the patient’s condition, leaving them vulnerable to secondary infections, and necessitating an effective second-line treatment option [5]. Generally, conventional cancer treatment approaches include surgical resection, followed by chemotherapy and/or radiation therapy, which are far from optimal [4]. Chemotherapy has been limited in its application, primarily due to issues surrounding non-specific cytotoxicity in healthy tissue, the inability to maintain the required therapeutic concentrations at the tumour site and the development of multiple drug resistance [6,7]. This warrants the formulation of a delivery system that can successfully transport a therapeutic agent to the tumor site with limited side effects on healthy tissue. An ideal delivery system must identify the target cells and release their therapeutic payload in a controlled manner over a predetermined period, while evading biological barriers, including clearance from the body.

Nanotechnology involves the study and manipulation of material at the nanoscale (<100 nm) and incorporates chemistry, medicine, engineering, biology, physics, and material science [8]. Various nanoparticles (NPs) have been investigated for their potential biomedical applications, with noble metal NPs showing promise due to their intrinsic physicochemical properties, including high biocompatibility, bioavailability, and low toxicity. In addition, their ease of synthesis and surface modification has made them an ideal candidate to function as a drug delivery vehicle [9,10]. Besides gold NPs (AuNPs), palladium NPs (PdNPs) are gaining popularity due to their inert core, low density, and similar physicochemical properties to AuNPs [11]. The combination of Au and Pd in the form of a bimetallic NP (BNP) is an attractive strategy for a delivery system. This is primarily due to the combined and enhanced physicochemical characteristics compared to their monometallic counterparts. Previous studies have shown that Au exhibits excellent biocompatibility and structural uniformity, in addition to being easily modified on its surface. In contrast, Pd has demonstrated enhanced catalytic activity due to its higher electron density at the NP interface. Studies involving noble metal-based bimetallic systems strongly indicate that core-shell conformations can result in synergistic optical, catalytic and biological responses that are not observed in monometallic systems [12,13,14,15]. These characteristics can be modified to enhance drug loading and ligand binding, ultimately improving intracellular trafficking. Considering the low interest in BNPs, the potential application of AuPd NPs as a dual-drug transferrin-targeted delivery system for cervical cancer therapy has been largely overlooked, thereby highlighting the novelty of this study. Stable BNPs ranging from 1–100 nm have been synthesized as monodispersed colloidal suspensions [16]. The long-term stability of these metal NPs can be improved by using suitable capping agents to prevent their aggregation in solution and extend the NP’s shelf life [11,17,18].

Cationic polymers, such as chitosan (CS), have been used in NP formulations to improve stability [11,19]. Additionally, CS is biocompatible, biodegradable, and non-toxic to cells, possessing free amino and hydroxyl groups that facilitate the binding of therapeutic agents and targeting ligands to the NP surface [20,21,22,23]. The encapsulation of chemotherapeutic drugs into CS enables a higher drug loading capacity, thereby enhancing the efficacy of the NP formulation [24]. The CS matrix also prevents degradation of the anticancer agents from environmental and physiological factors [25].

The broad-spectrum anticancer activity of doxorubicin (DOX) and 5-fluorouracil (5-FU) made them an obvious choice for this study [26]. Both drugs have demonstrated their efficiency as anticancer agents, effectively killing cancer tissue. 5-FU and DOX exhibited complementary mechanisms of action in cancer therapy. However, their non-specificity of action is a major limiting factor to their continued use [27,28]. Their primary mechanism of action involves targeting rapidly dividing cells, including those found in healthy tissue. This results in severe side effects commonly associated with chemotherapy [29,30]. Incorporating these cytotoxic agents into a targeted bimetallic drug delivery system enables cancer cell specificity while lowering non-specific cytotoxicity in healthy cells. Additionally, research has shown that this combination of chemotherapeutic drugs can lead to increased anticancer activity. 5-FU has demonstrated its ability to inhibit thymidylate synthase, thereby disrupting nucleotide synthesis. In comparison, DOX intercalates with DNA, inhibiting topoisomerase II. This action results in damage to the cell’s DNA, ultimately leading to cell death, also known as apoptosis. Studies have demonstrated that co-administration of these agents can enhance antiproliferative effects, increase apoptotic signalling and reduce the required dosage of each drug as different cellular mechanisms are targeted [31,32,33].

Research has highlighted the importance of designing drug delivery systems that can exploit the unique biochemical and physiological features of the tumor microenvironment [34]. A recent study highlighted a nanotherapeutic system that incorporates photodynamic, photothermal, and chemodynamic capabilities. Findings from this study reinforced the importance of designing adaptive nanostructures to improve the efficacy of therapeutic systems for cancer [35]. Hence, designing a nanosystem with structural, chemical and biological features tailored to tumor-specific conditions is essential. Within this context, Au–Pd NPs offer a complementary approach, combining the stability of noble metals with tuneable surfaces, drug-loading potential, and targeted delivery, supporting their relevance in cervical cancer therapy.

Targeted drug delivery systems have the potential to provide cell-specific cytotoxicity in vitro. Adding transferrin (Tf) into a nanocomplex has been shown to improve the efficacy of a delivery vehicle [36]. HeLa cells highly express Tf receptors on their surface, enabling active targeting by adding a Tf ligand to the NP surface [37,38]. This promotes internalization through receptor-mediated endocytosis [37]. Currently, research focusing utilizing AuPd BNPs in dual-drug delivery is very limited. In addition, similar Tf-targeted AuPd BNPs have not been explored in cancer therapeutics. This study aimed to synthesize, characterize, and investigate the potential of a Tf-targeted AuPd BNP dual-drug delivery system in vitro. Their drug release profiles and anticancer activities in human cells were assessed using the cervical carcinoma (HeLa), breast adenocarcinoma (MCF-7), and embryonic kidney (HEK293) cells, noting dose dependency and cell specificity, with a particular focus on the HeLa cells. The potential of BNPs in the targeted delivery of anticancer agents in vitro is yet to be fully explored, compared to their monometallic counterparts.

## 2. Results

### 2.1. UV-Visible (UV-Vis) Spectroscopy Studies

**Figure 1:**
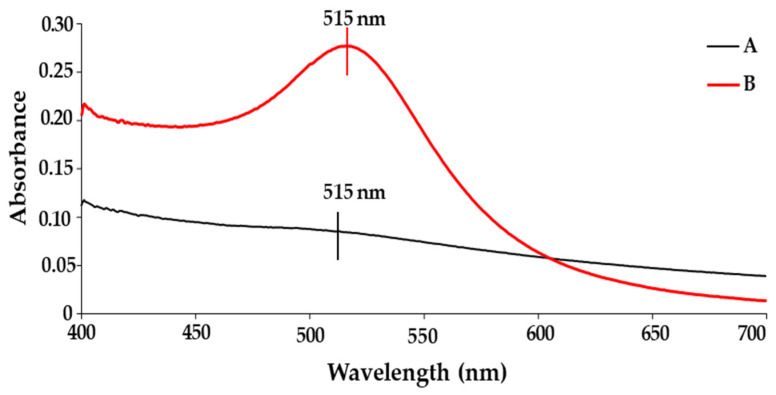
UV-visible spectra of (A) AuPd NP and (B) AuNP.

UV-vis spectroscopy is commonly used to identify absorbance peaks corresponding to a NP’s surface plasmon resonance (SPR). AuNPs have a peak in the range of 520–540 nm for spherical NPs. A red or blue shift in the UV-vis spectrum indicates successful conjugation to polymers, drugs, or other significant changes in the surface chemistry of the NP. It has been well-documented that Pd does not possess a well-defined peak; however, the complete reduction in Pd(II) ions was confirmed by the absence of a peak above 300 nm [39]. A study by Ho et al. (2004) demonstrated similar results during thermal reduction in Pd [40]. The presence of Pd on the surface of the BNP will dampen the expected peak of AuNP, which forms the core of the BNP. This is supported by data obtained from UV-vis analysis of the BNPs (Figure 1), where the AuNP peak is visible, albeit faintly. These results correlate well with the literature, TEM and XRD, confirming that the BNPs of core-shell conformation were successfully synthesised.

### 2.2. ICP-OES and FTIR Analysis

ICP analysis confirmed the concentration of synthesised Au-core Pd-shell BNP to be 3.97 mg/L for Au and 3.72 mg/L for Pd.

**Figure 2:**
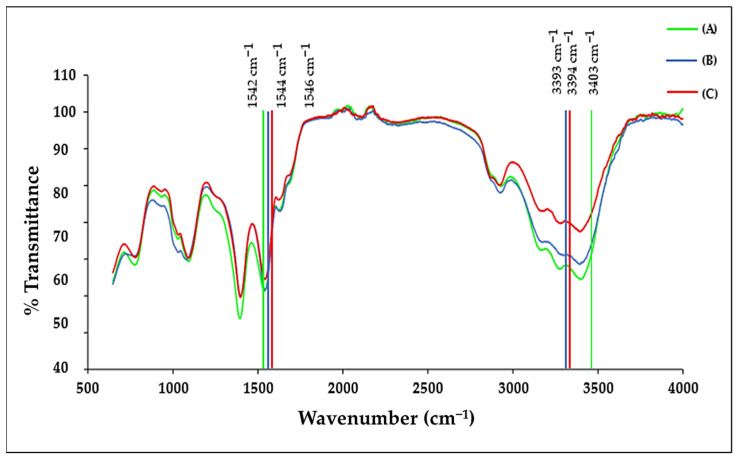
The FTIR spectrum of (A) 5-FU-CS-Tf-BNPs; (B) DOX-CS-Tf-BNPs, and (C) Dual Drug-CS-Tf-BNPs.

The FTIR spectra, as seen in Figure 2 for the functionalised BNPs, displayed the characteristic vibrational bands of citrate-capped Au-Pd NPs together with the expected bands for Tf, CS, 5-FU and DOX. The observed peak shifts following NP conjugation indicated changes in the structure of these molecules. The respective bimetallic nanocomplexes containing 5-FU, DOX, and dual drug-CS-Tf displayed a wide band in the 3000–3600 cm^−1^ region (Figure 2).

Peaks were visible at 3403 cm^−1^, 3393 cm^−1^, and 3394 cm^−1^ for the drug-containing BNPs (5-FU, DOX, and dual-drug). These values correspond to an O–H stretch observed in CS. The characteristic peak at 1648 cm^−1^ of CS corresponds to the amide group, which shifted to 1542 cm^−1^, 1546 cm^−1^, and 1544 cm^−1^ for the respective BNPs containing 5-FU, DOX and the dual-drug [41]. Tf had a strong absorption peak at 1635 cm^−1^, overlapping the amide I absorption band. This corresponded to the stretching vibration of histidine. Under normal conditions, aspartic acid strongly absorbs at 1402 cm^−1^ due to symmetric stretching vibrations of the carbonyl group [42]. However, in coordination with a metal ion, the band has been known to shift [43]. Tf displayed a strong absorption peak at 1392 cm^−1^ for all targeted BNP nanocomplexes, with a downshift of ~10 cm^−1^. The shifts in peaks indicate the presence of the -COOH group of aspartic acid in Tf [44].

The anticancer drug, 5-FU, displayed a characteristic C-H stretch in the 2800 cm^−1^ to 3100 cm^−1^ region, which can be attributed to the vibration of the imide stretch. The C=C and C=N stretching vibrations of 5-FU can be attributed to the absorbance bands in the region of 1431 cm^−1^ to 1658 cm^−1^, while the bands in the region of 1398 cm^−1^ are due to the vibration of the aromatic ring [45]. 5-FU has characteristic peaks in the fingerprint region of the spectra, which were significantly reduced in the targeted complexes of the BNPs [46]. This suggests that the drug was encapsulated within the CS matrix. DOX displays characteristic peaks at 3382 cm^−1^ and 1618 cm^−1^ [47,48], and upon CS encapsulation and binding to the BNPs, the peak shifted to 3393 cm^−1^, resulting in the O–H stretch present in CS increasing in intensity. There was also a shift from 1618 cm^−1^ to 1636 cm^−1^ and 1632 cm^−1^ [49]. These observed shifts were due to the negatively charged carboxylate groups on DOX and the positively charged amino groups on CS interacting with each other. The characteristic bands of CS and Tf were present in the respective bimetallic nanocomplexes. These minor shifts provided confirmation of the successful encapsulation of the drugs by the NPs. Table 1 lists the significant characteristic peaks for the polymers and NPs.

### 2.3. X-Ray Diffraction (XRD)

**Figure 3:**
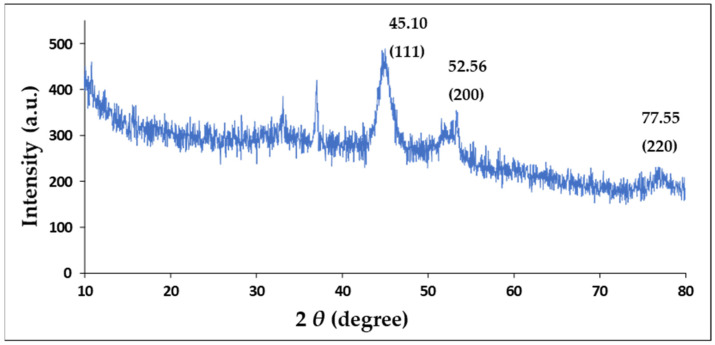
X-Ray Diffraction spectrum of the AuPd BNPs.

XRD was used to confirm the crystal structure of the Au-core Pd-shell BNPs. The XRD pattern (Figure 3) exhibits four peaks at 2θ angles of 45.10°, 52.56°, 77.54°, and 94.50°, corresponding to the Bragg reflections of (111), (200), (220), and (311), respectively. The positions of the peaks suggested a face-centred cubic (FCC) crystal structure of AuPd BNP, exhibiting a core-shell formation with a cubic close-packed (CCP) atom arrangement in an octahedral void, rather than mixed oxides or other impurity phases [50]. XRD analysis confirmed that the BNP comprises Au and Pd, as per cross-referencing by the International Center for Diffraction Data files ICSD:180875 and the International Crystal Structure Database file PaN ICSD:98-108-0875. Lattice parameters were calculated as a = b = c = 4.0400 Å (0.404 nm) from XRD measurements. The average crystallite size, calculated using the Debye-Scherrer equation, was approximately 66 Å (6.6 nm). These results, in addition to the lattice spacing values identified in high-resolution TEM (Section 2.4), indicate that the synthesis of Au-Pd NPs with a core-shell conformation was successful. Table 2 provides details on the important peaks in the XRD spectrum.

### 2.4. Transmission Electron Microscopy (TEM)

**Figure 4:**
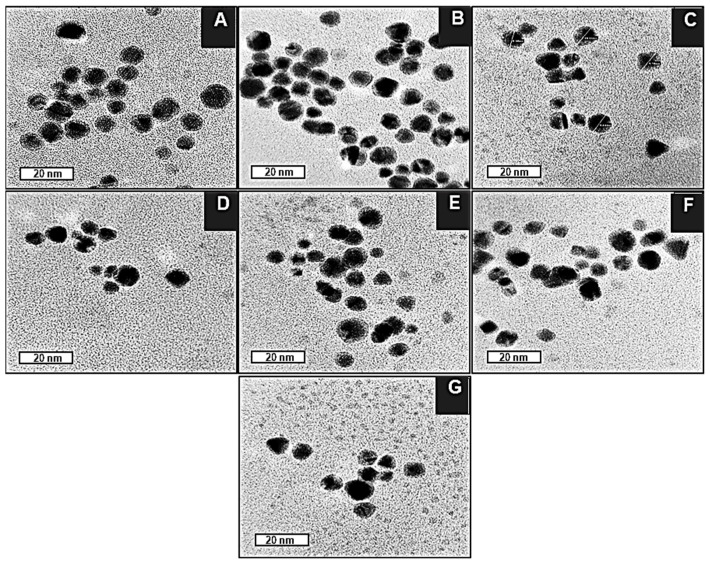
TEM images of (**A**) BNP, (**B**) 5-FU-CS-BNP, (**C**) DOX-CS-BNP, (**D**) Dual Drug-CS-BNP, (**E**) 5-FU-CS-Tf-BNP (**F**) DOX-CS-Tf-BNP, (**G**) Dual Drug-CS-Tf-BNP. Bar = 20 nm.

**Figure 5:**
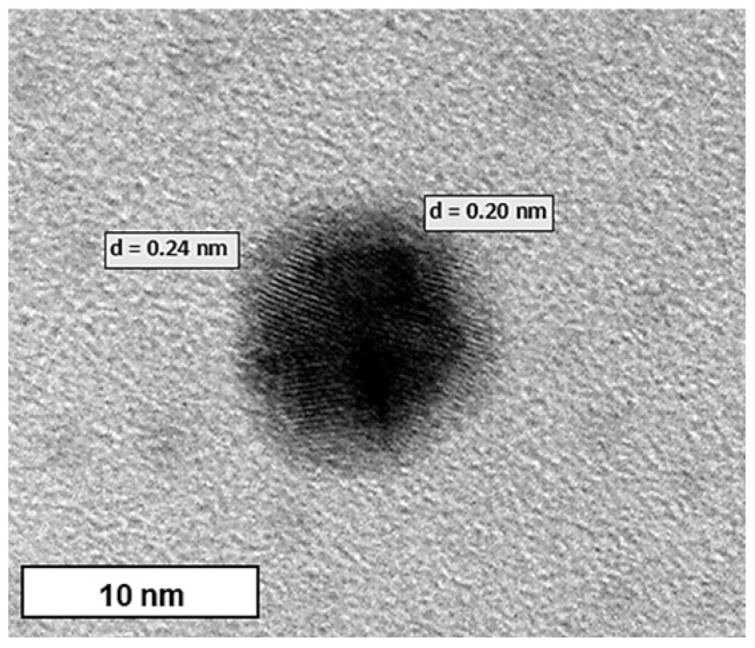
HRTEM image of a BNP lattice fringes with d-spacing. Bar = 10 nm.

TEM images of the BNPs (Figure 4A) revealed them to be spherical and uniform in shape, with a good degree of polydispersity. The core-shell conformation of the BNPs can be seen as a dense Au core surrounded by a less dense Pd shell. This correlates with Figure 5, in which the d-spacing of the core is approximately 0.24 nm, corresponding to the (111) planes of FCC Au, as determined by XRD [51]. In contrast, the d-spacing of the bimetallic shell is 0.20, which is representative of the (200) crystal plane of cubic close-packed Pd [52]. These results corresponded well with data from XRD analysis, confirming that the BNPs have been successfully synthesized. For the nanocomplexes (Figure 4B–G), 5-FU-CS-BNP; DOX-CS-BNP; dual drug-CS-BNP; 5-FU-CS-Tf-BNP; DOX-CS-Tf-BNP, and dual drug-CS-Tf-BNP, conjugation of 5-FU- and DOX-CS and Tf did not have a significant impact on the polydispersity of the solution. The NPs remained well dispersed, with low levels of aggregation, indicating good colloidal stability.

Overall, these TEM results correlate with those from NTA, FTIR, and XRD, highlighting the potential of these BNPs as drug nanocarriers due to their favourable size, shape, and stability.

### 2.5. Nanoparticle Tracking Analysis (NTA)

Table 3 displays the NTA analysis of the BNPs and their respective nanocomplexes. The BNPs had a hydrodynamic diameter of 73.4 ± 9.8 nm with a zeta potential (ZP) of −18.2 ± 2.5 mV, indicating that the synthesized NPs were stable. The ZP shifted to +16.1 mV for the 5-FU encapsulated BNP and +15.4 mV for the DOX encapsulated BNP, respectively. The ZP of 5-FU encapsulated CS-Tf-BNP, DOX encapsulated CS-Tf-BNP and dual drug encapsulated CS-Tf-BNP was recorded at +15.4 mV, +12.8 mV and +16.9 mV, respectively. This positive shift in ZP from the BNPs to the respective nanocomplexes suggested a strong and favorable association between NP surface and the constituents, indicating good colloidal stability. This data correlates with the results obtained from the FTIR and TEM studies. The PDI values (Table 3) remained low (<0.2) for the BNP nanocomplexes. The narrow range of PDI values indicates a more uniform colloidal solution with a smaller size distribution, making it a suitable candidate for drug delivery systems. The long-term stability of the BNPs was determined through stability studies (Table 4).

The BNPs displayed good long-term stability throughout the study, which is an ideal attribute for a drug delivery system. On day 0, the BNPs had a size of 73.4 nm with a ZP of −28.7 mV. On day 28, the size of the BNPs decreased to 61.5 nm while their ZP improved to −27.3 mV. However, on the last day of the study (after 30 weeks), the size of the BNPs increased slightly to 76.01 nm but with an excellent ZP of −69.27 mV. Overall, the BNPs have demonstrated improved stability over time. The results, as reflected in Table 3 and Table 4, strongly suggest reorganisation at the surface of the citrate-stabilised BNP, as opposed to degradation of the synthesized NPs. Citrate and other similar small anionic capping agents possess the ability to slowly redistribute on the surface of the NP. This reorganisation can have a direct impact on the surface coverage of the capping agent, the adsorption geometry, and ion pairing with counterions in solution. Over an extended period, this has the potential to improve the effective surface charge density. This correlates well with the observed increase in zeta potential over the study period [53]. In bimetallic systems, gradual surface reconstruction has been reported during storage or mild ageing, which can affect surface composition [54]. These results further support the hypothesis that BNPs can serve as efficient delivery vehicles.

### 2.6. Encapsulation Efficiency

The encapsulation efficiency of 5-FU and DOX was calculated using the ratio of free drug to bound drug. The encapsulation efficiency of DOX and 5-FU was determined to be 74.81% and 72.63%, respectively.

### 2.7. Drug Release and Kinetics Studies

**Figure 6:**
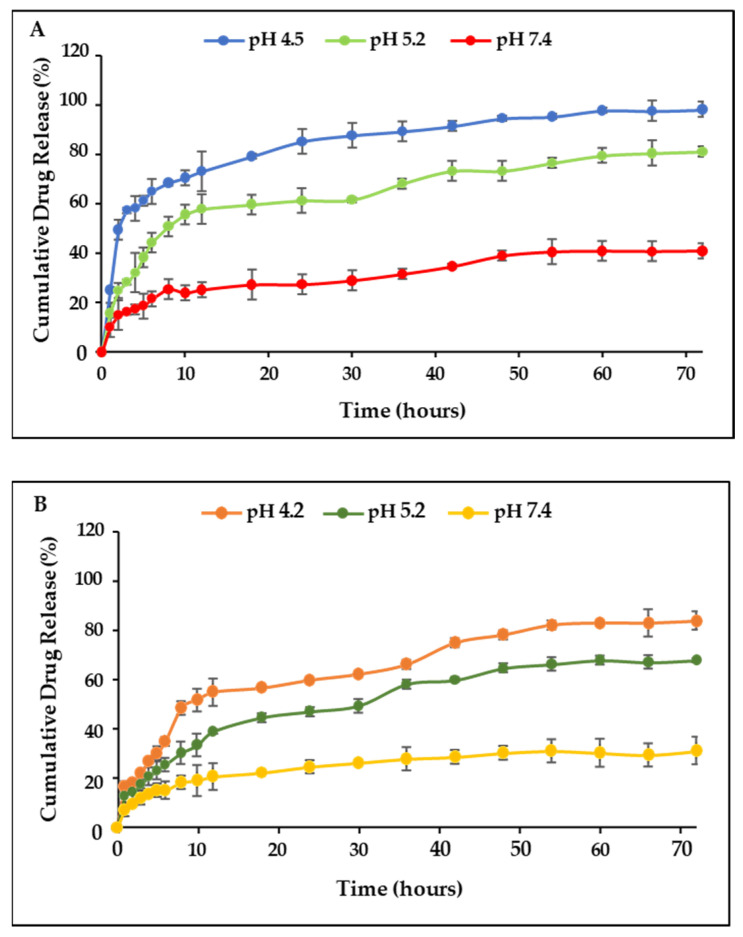
The drug release profile of (**A**) DOX-CS-Tf-BNPs and (**B**) 5-FU-CS-Tf-BNPs at pH levels of 4.2, 5.2, and 7.4.

**Figure 7:**
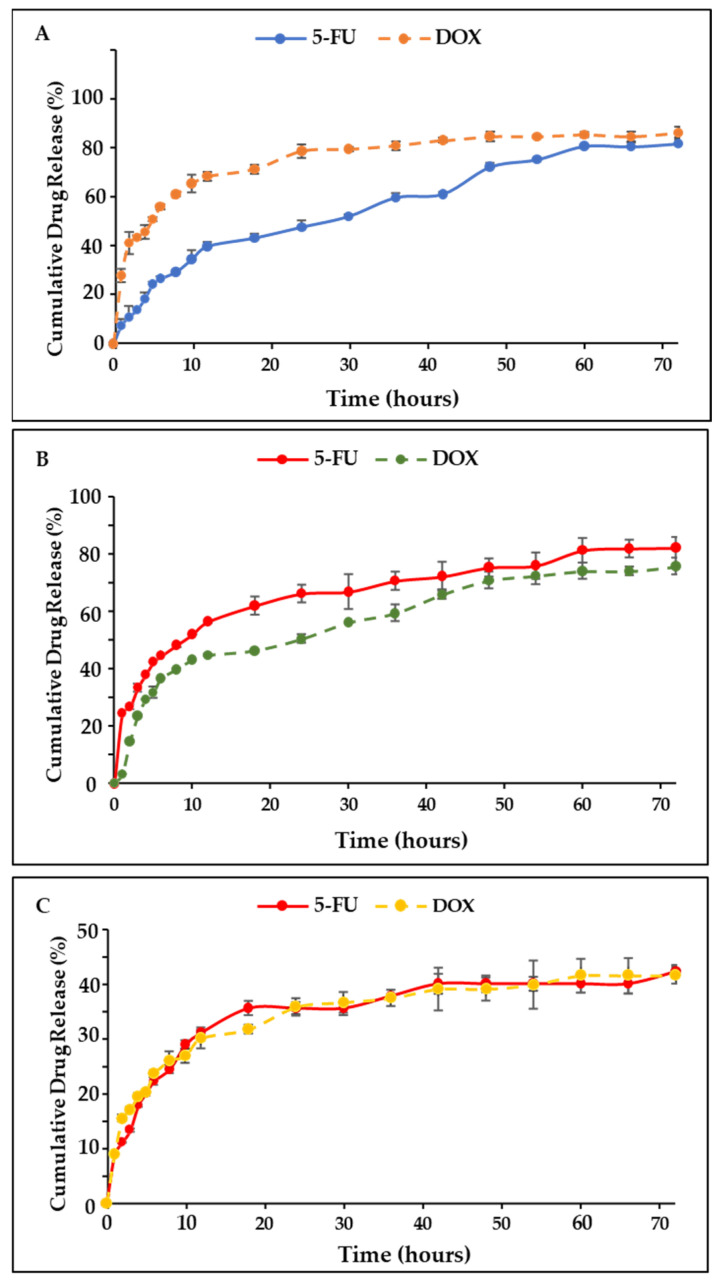
The drug release profile of the dual drug-CS-Tf-BNPs at (**A**) pH 4.2, (**B**) pH 5.2 and (**C**) pH 7.4. BNP nanocomplexes yielded high cumulative release, with release profiles being more controlled.

The drug release profiles of 5-FU-CS-Tf-BNPs and DOX-CS-Tf-BNPs are shown in Figure 6. The nanocomplex exhibited the highest cumulative release of 5-FU (98.39%) at a pH of 4.2, which is similar to the pH of a tumor microenvironment. The release at pH 5.2 and 7.4 was 81.35% and 41.05%, respectively. At pH 4.2, 91.03% of DOX was released, with 73.91% and 33.91% released at pH 5.2 and 7.4. The dual drug-CS-Tf-BNP (Figure 7) displayed a similar pattern of release at pH 4.2 (90.22% of 5-FU and 95.12% of DOX) but a decrease at pH 5.2 (82.20% of 5-FU and 75.61% of DOX). At pH 7.4, less than 50% of 5-FU and DOX was released.

The pH-dependent release behaviour observed for the BNP nanocomplexes is consistent with similar pH-responsive NP systems, which have been designed for tumour-targeted drug delivery. Several systems, including citrate-stabilised noble metal NPs, polymer-coated nanostructures and metal hybrid systems, have shown improved drug release under acidic conditions. This is primarily due to the protonation and resultant changes in ligand conformation, leading to the release of the therapeutic agents [55,56,57]. Literature indicates that the tumor microenvironment typically exhibits pH values between 5.5 and 6.5, which can result in faster diffusion of weakly bound therapeutic molecules from NPs. The enhanced release of 5-FU and DOX under acidic conditions, observed for the BNPs nanocomplexes, aligns well with the literature.

Data from the drug release was fitted into suitable mathematical models. Table 5 and Table 6 display the release kinetics of the 5-FU-CS-Tf-BNP nanocomplexes at pH levels of 4.2, 5.2, and 7.4. The release of 5-FU fitted the Higuchi’s (R2 = 0.963) model at pH 4.2. This is a strong indication that the drug’s release occurred due to diffusion from the nanocomplex. The Kopcha’s model (A > AB, R2 = 0.9173) was a good fit for the data and correlated well with the findings, confirming that release occurred through diffusion. At pH levels of 5.2 and 7.4, the release of 5-FU fitted Higuchi’s model (R2 = 0.9602) and (R2 = 0.9375), respectively, with the Kopcha model correlating at pH 5.2 (A > B, R2 = 0.9879) and 7.4 (A > B, R2 = 0.9884), supporting the claim that release occurred through diffusion.

Table 7 and Table 8 display the release kinetics of the DOX-CS-Tf-BNP nanocomplexes. The release of DOX at pH 4.2 was best described by the First Order (R2 = 0.9694) release model. According to this model, the rate of drug release is proportional to the amount of drug present in the delivery system at any time. DOX release at pH 5.2, fitted Higuchi’s model best (R2 = 0.9782) and the Kopcha model (A > B, R2 = 0.9728), indicating that the release was primarily due to diffusion over erosion. At a pH of 7.4, the release fitted Higuchi’s (R2 = 0.926) and Kopcha’s (A > B, R2 = 0.9989) models, confirming that release was more due to diffusion than erosion.

Table 9 and Table 10 display the correlation coefficients, release exponents and diffusion and erosion constants of the dual drug-CS-Tf-BNPs. 5-FU release at pH 4.2, the resembled Higuchi’s release model (R2 = 0.9734), which indicated that diffusion was the dominant factor in the release of the drug. This was substantiated by Kopcha’s release mechanism (A > AB, R2 = 0.923). According to the Korsmeyer-Peppas model (*n* = 0.58, R2 = 0.7554), the release was characterised by anomalous diffusion. The diffusion of DOX at pH 4.2 also followed a similar release mechanism, with Higuchi’s model (R2 = 0.9604) being the best fit, with Kopcha’s release mechanism (A > AB, R2 = 0.9785) indicating that diffusion occurred at a higher rate over erosion.

The release of DOX at a pH of 5.2 was in line with Higuchi’s model (R2 = 0.8996), while Kopcha’s model again showed that diffusion was dominant over erosion (A > AB, R2 = 0.9826). The release of DOX at pH 7.4 followed the Korsmeyer-Peppas model (*n* = 0.31, R2 = 0.9875). This suggested that DOX release was due to the polymer matrix undergoing stress and swelling. The release exponent indicated that Quasi-Fickian diffusion occurred, with Kopach’s model confirming that the DOX release was primarily due to diffusion over erosion. The Kopcha model indicated that drug release from the BNP nanocomplexes was predominantly diffusion-controlled, suggesting a gradual and sustained release of the encapsulated drugs. This observation aligns with previous reports in which diffusion-driven release supported prolonged drug availability at the tumour site while limiting premature drug loss during circulation [58]. At acidic pH, the enhanced molecular mobility, swelling of the CS polymer and increased solubility of the drugs further promoted their release. Sustained diffusion from metal-based nanocarriers has been associated with improved therapeutic indices and reduced toxicity in normal cells [59,60]. These findings indicate that the release kinetics demonstrated by the BNP nanocomplexes may favour tumour-selective drug accumulation and enhance therapeutic efficacy in vivo.

### 2.8. In Vitro Cytotoxicity

The MTT or 3-(4,5-dimethylthiazol-2-yl)-2,5-diphenyl tetrazolium bromide assay relies on reduction in tetrazolium salts to their intensely colored formazan product [61]. This provides a good estimation of the metabolic activity of a population of cells and is often used to determine the cytotoxicity of NPs and nanocomplexes [62]. In this study, the cytotoxicity profile of the BNPs and their respective nanocomplexes was evaluated using the ratios listed in Table 11. The concentrations of free 5-FU and DOX (controls) were determined from their concentrations within the CS-encapsulated nanocomplexes (2.60 × 10^−4^ mM 5-FU; 5.86 × 10^−2^ mM DOX) and the previously calculated encapsulation efficiency.

**Figure 8:**
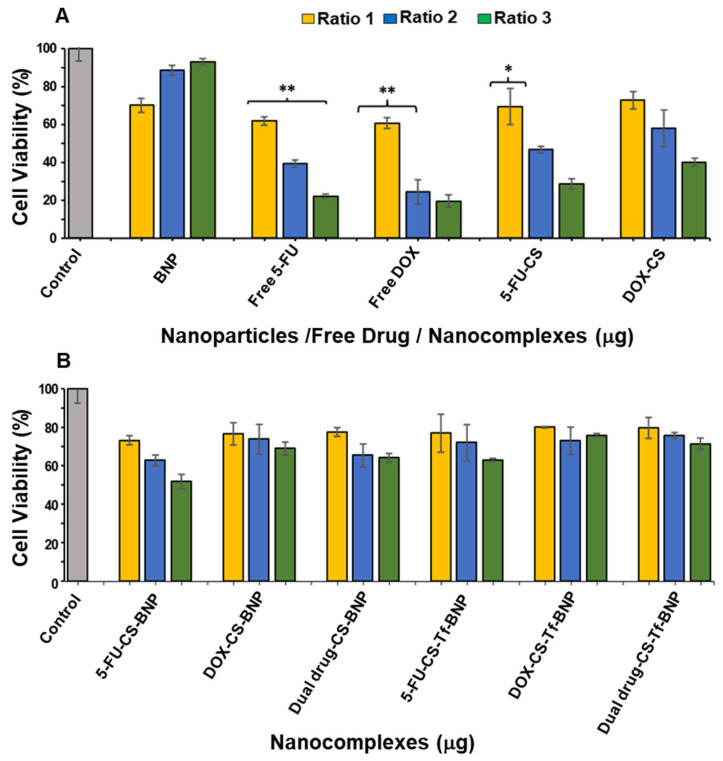
Cytotoxicity assay of (**A**) BNPs, Free 5-FU, Free DOX, 5-FU-CS and DOX-CS; and (**B**) 5 5-FU-CS-BNP, DOX-CS-BNP; dual drug-CS-BNP, 5-FU-CS-Tf-BNP, DOX-CS-Tf-BNP and dual drug-CS-Tf-BNP in the HEK293 cells. The cells were treated with nanocomplexes at 3 ratios (Table 11). Data are represented as means ± SD (*n* = 3); * *p* < 0.05 and ** *p* < 0.01 were considered statistically significant.

**Figure 9:**
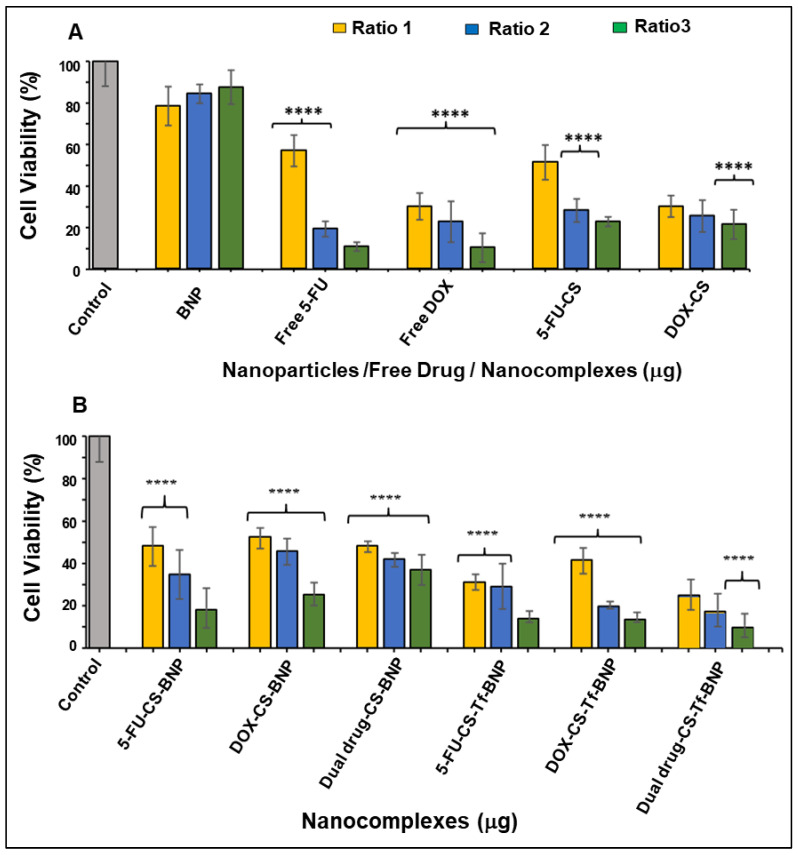
Cytotoxicity assay of (**A**) BNP, Free 5-FU, Free DOX, 5-FU-CS and DOX-CS; (**B**) FU-CS-BNP, DOX-CS-BNP, dual drug-CS-BNP, 5-FU-CS-Tf-BNP, DOX-CS-Tf-BNP and dual drug-CS-Tf-BNP in the HeLa cells. Cells were treated with nanocomplexes at 3 ratios (Table 10). Data are represented as means ± SD (*n* = 3); **** *p* < 0.0001 was considered statistically significant.

**Figure 10:**
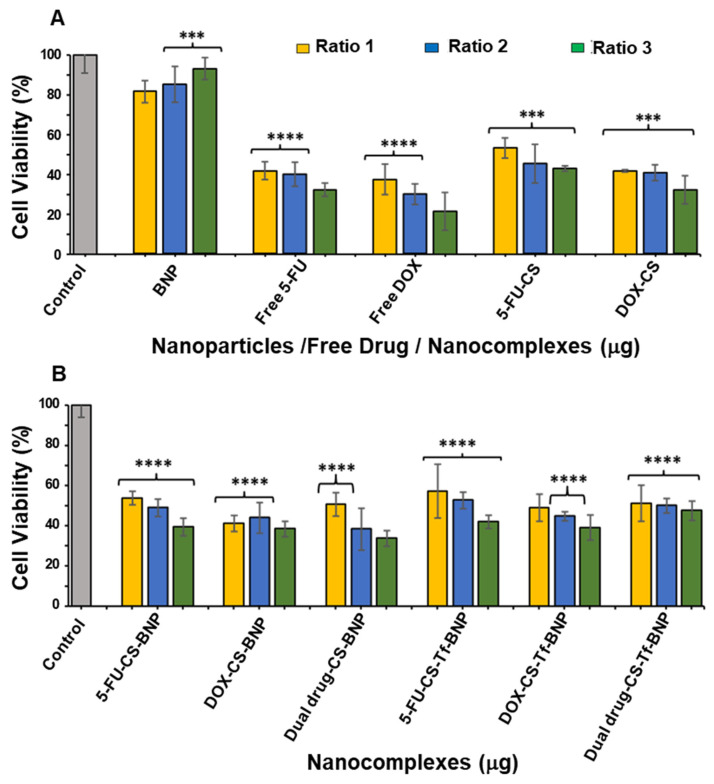
Cytotoxicity assay of (**A**) BNP, Free 5-FU, Free DOX, 5-FU-CS and DOX-CS; and (**B**) 5-FU-CS-BNP, DOX-CS-BNP; dual drug-CS-BNP, 5-FU-CS-Tf-BNP, DOX-CS-Tf-BNP and dual drug-CS-Tf-BNP in the MCF-7 cells. Cells were treated with nanocomplexes at 3 ratios (Table 10). Data are represented as means ± SD (*n* = 3); *** *p* < 0.001, **** *p* < 0.0001 were considered statistically significant.

The cytotoxicity profile of the BNPs and their nanocomplexes is displayed in Figure 8, Figure 9 and Figure 10. Similar trends in cytotoxicity were observed in all cells. The BNPs alone exhibited little to no cytotoxicity in HEK293, HeLa, and MCF-7 cells, with improved cell viability noted at higher concentrations, similar to that reported for noble metal NPs [63,64]. The presence of the targeting ligand Tf had a unique effect on each cell line. There was an overall improvement in cytotoxicity in HEK293 cells when treated with the Tf-targeted nanocomplex, whereas HeLa and MCF-7 cells showed a decrease in cell viability.

HEK293 cells were used as a control normal cell line. Treatment of the HEK293 cells with the BNPs (Figure 8) revealed that the free 5-FU and DOX generated the lowest cell viability of 22.20% and 19.57%, respectively. The 5-FU encapsulated CS and DOX encapsulated CS generated improved cell viabilities of 28.61% and 40.13%, indicating that the method of encapsulation with CS directly affected the release pattern of the respective drugs. The cell viabilities using 5-FU-CS-BNP, DOX-CS-BNP and dual drug-CS-BNP were significantly improved and were 51.64%, 68.91% and 64.14%, respectively. The addition of Tf to the NP improved cell viability to 62.99%, 75.82%, and 71.38%, respectively. These results represent a considerable improvement in cell viability for the BNP nanocomplexes, highlighting the advantage of using BNPs as drug-delivery systems.

The cytotoxicity levels in the HeLa cells are shown in Figure 9. Tf-targeted nanocomplexes displayed dose-dependent cytotoxicity. The HeLa cells treated with 5-FU-CS-BNPs, DOX-CS-BNPs, and dual drug-CS-BNPs had the lowest cell viabilities, at 18.15%, 25.09%, and 36.90%, respectively. For the targeted nanocomplexes of 5-FU-CS-Tf-BNPs, DOX-CS-Tf-BNPs and dual drug-CS-Tf-BNPs, the cell viabilities were 13.91%, 13.65% and 9.58%, respectively. This high cell death could have been due to the presence of Tf in the NP and the high levels of TfR on the HeLa cell surface. This resulted in greater cellular uptake.

Free 5-FU and DOX exhibited greater cytotoxicity than the BNPs in the MCF-7 cells, which was to be expected, as both 5-FU and DOX are potent cytotoxic agents in cancer cells (Figure 10). A similar trend of dose-dependent cytotoxicity was observed for all untargeted nanocomplexes, with the targeted nanocomplexes (5-FU-CS-Tf-BNPs, DOX-CS-Tf-BNPs, and dual drug-CS-Tf-BNPs), generating cell viabilities of 39.38%, 38.36%, and 33.62%, respectively (Figure 10B). Here again, the presence of Tf directly impacted cell viability.

### 2.9. Receptor Binding Competition Study

This assay determined if the Tf-containing nanocomplexes (5-FU-CS-Tf-BNPs, DOX-CS-Tf-BNPs, and dual drug-CS-Tf-BNPs) were taken up by the HeLa cells via the Tf receptor (TfR). HeLa cells were pre-incubated with excess Tf before treatment with the nanocomplexes to enable the Tf molecules to bind to the Tf receptors on the surface of the HeLa cells [37]. This binding should block or limit the number of free receptors that could facilitate the uptake of the Tf-targeted nanocomplexes.

**Figure 11:**
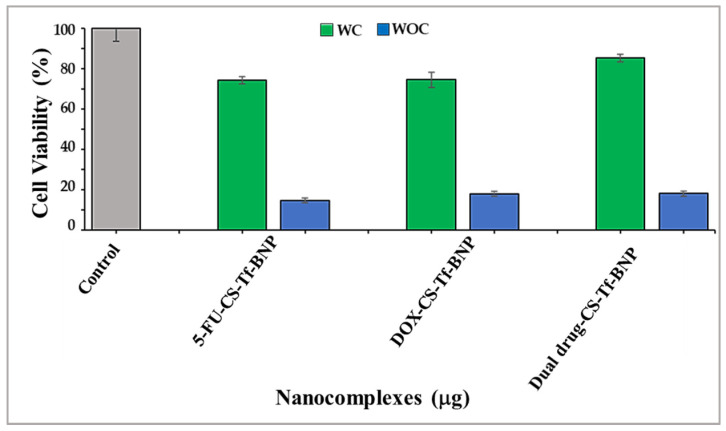
Receptor binding competition assay for 5-FU-CS-Tf-BNPs, DOX-CS-Tf-BNPs, and dual drug-CS-Tf-BNPs, treated with a Tf competitor (WC) and without a Tf competitor (WOC) in HeLa cells. Competitor = free Tf. Cells were treated with nanocomplexes at ratio 3 (Table 10). Data are represented as means ± SD (*n* = 3).

The cytotoxicity study of Tf-targeted BNPs (Figure 11) revealed that cells exposed to the Tf ligand prior to treatment with the nanocomplexes exhibited cell viabilities ranging from 74% to 85%. There was a drastic decrease in cell viability for cells with no prior Tf treatment. These cells exhibited cell viabilities of less than 18%. This study highlights that the enhanced anticancer activity of the Tf-targeted nanocomplexes can only be attributed to the presence of Tf on the surface of the BNP. Overall, the Tf-based BNPs resulted in significantly lower cell viability in the Tf-receptor-rich HeLa cells. This suggests their potential as suitable vehicles for targeted delivery in cancer treatment. Although Tf expression is significantly higher in HeLa, these receptors are also present, at lower levels, in certain normal tissues. The physiological expression of these receptors has been reported in erythroid precursors, intestinal crypt cells, activated lymphocytes, and hepatocytes, where regulated iron uptake is essential for metabolic activity [65,66]. Studies have indicated the possibility of limited off-target uptake in tissues with moderate Tf activity. In vivo studies using Tf-functionalised metal and polymer nanocarriers have shown preferential tumour accumulation, but some distribution to the liver and spleen has also been reported [67]. The overall accumulation in the tumour environment, compared to other tissues, indicates that Tf-mediated targeting can achieve meaningful selectivity.

### 2.10. Intracellular Oxidative Stress Analysis

**Figure 12:**
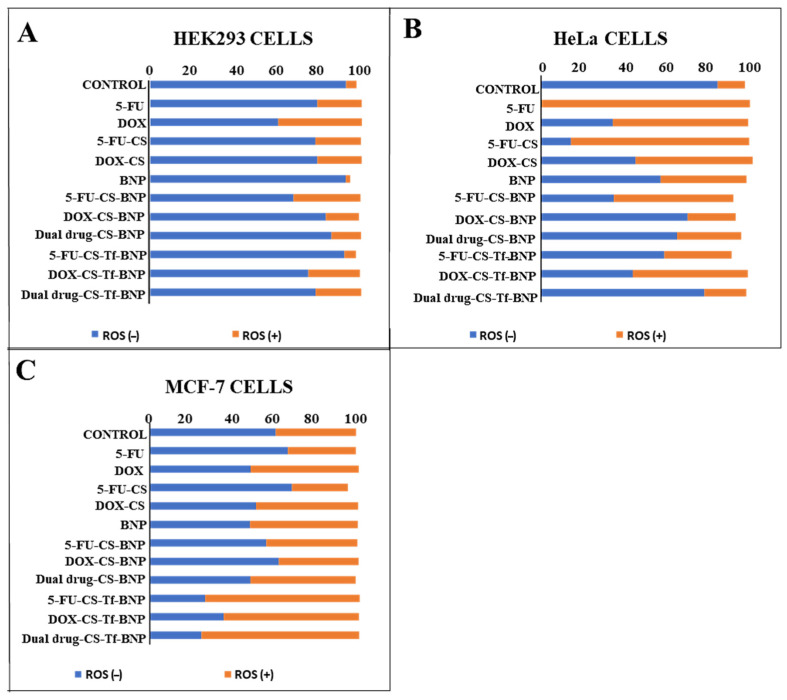
Oxidative stress generated by free 5-FU, free DOX, 5-FU-CS, DOX-CS and the BNPs and their nanocomplexes in the (**A**) HEK293, (**B**) HeLa and (**C**) MCF-7 cells. Nanocomplexes were used at their IC_50_ concentrations (Table 11).

HEK293, HeLa and MCF-7 cells were treated with BNP nanocomplexes (Figure 12) at amounts set out in Table 12. The BNPs generated a higher percentage of ROS (−) species (>90%) in HEK293 cells (Figure 12A), highlighting the innate ability of the BNPs to reduce oxidative stress in non-cancerous cells. The HeLa cells (Figure 12B) were more resistant to oxidative stress, while the MCF-7 cells (Figure 12C) exhibited higher levels of ROS (+) cell population. These results support the hypothesis that the 5-FU- and DOX-CS-BNPs, as well as the Tf nanocomplexes, exert cytotoxic effects on HeLa cells through an alternative pathway. The higher ROS levels observed in the HeLa cells treated with the Tf-targeted Au–Pd nanocomplexes corresponded with the lower IC_50_ values recorded for this cell line. Increased ROS production is known to induce mitochondrial dysfunction, DNA damage, and activation of apoptotic pathways, enhancing cytotoxicity in tumour cells [68]. In contrast, the comparatively lower ROS generation in HEK293 cells correlated well with their reduced Tf receptor expression and higher IC_50_ values, suggesting less efficient NP uptake and reduced susceptibility to treatment.

The elevated ROS levels and enhanced apoptotic features observed in the HeLa and MCF-7 cells treated with the CS-Tf-BNP nanocomplexes are consistent with the mechanisms of action of 5-FU and DOX. DOX is known to undergo redox cycling in the presence of intracellular iron, generating superoxide anions and hydroxyl radicals that contribute to mitochondrial membrane damage, DNA strand breaks, and activation of the caspase system [69,70]. 5-FU has been similarly shown to induce oxidative stress through the disruption of nucleotide synthesis, misincorporation into RNA, and activation of cellular stress response pathways, resulting in mitochondrial disruption and apoptosis [71].

### 2.11. Fluorescent Apoptosis Studies

Fluorescent dual-dye-based apoptotic studies further confirmed the observed cytotoxicity in the cells and were used to determine if apoptosis was responsible for cell death. This technique utilizes a fluorescent-based dual dye system made up of acridine orange and ethidium bromide (AO/EB) to detect live, early apoptotic, late apoptotic, and necrotic cells [72].

**Figure 13:**
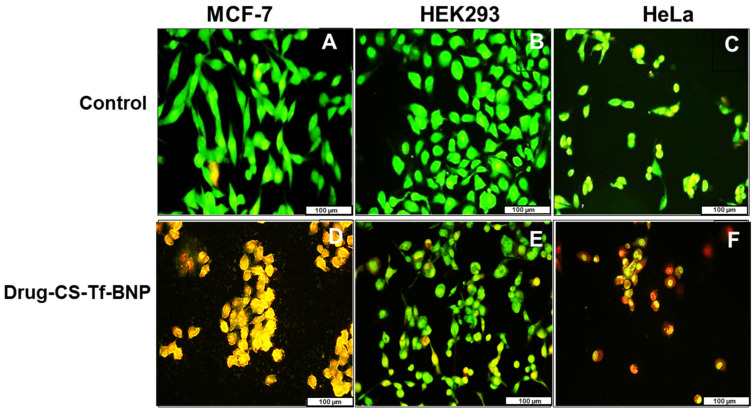
Fluorescent mages (20× magnification) of (**A**) MCF, (**B**) HEK293 and (**C**) HeLa cell controls and (**D**) MCF, (**E**) HEK293 and (**F**) HeLa cells treated with dual drug-CS-Tf-BNPs. The scale bar represents 100 µm.

Apoptotic features were visible in all treated cells (Figure 13), with the Tf-targeted nanocomplexes demonstrating a more significant induction of apoptosis in the cancer cells, as confirmed by the apoptotic indices (Table 13). The Tf-nano complexes are reflected in Figure 13 as they produced the best anticancer activity. The HEK293 cells produced lower apoptotic indices than the cancer cells. These results correlate well with the cell viability seen in the cytotoxicity study.

## 3. Discussion

This study aimed to develop a Tf-targeted, dual anticancer drug-loaded BNP delivery system. The system was designed to exhibit a well-defined core-shell formation, with favourable physicochemical characteristics, including size, shape, and stability, that selectively release anticancer agents in the acidic tumour microenvironment of cervical cancer cells through the incorporation of a pH-sensitive encapsulating polymer. Analysis of the physicochemical properties confirmed that the Au–Pd BMNPs and their respective nanocomplexes possessed desirable characteristics for a potential drug delivery system. Results from UV–vis and FTIR spectroscopy, as well as ICP and XRD data, confirmed and correlated well with each other regarding the successful formation of Au-Pd BNPs. Specifically, the broadening and subsequent damping of the characteristic Au SPR peak, together with the absence of a distinct Pd band above 300 nm, is consistent with recent literature on Au-Pd BNP systems. The ratio of metals within a bimetallic plays a vital role in forming a peak [73], in addition to affecting their physicochemical properties and antitumor activity [74]. Au and Pd were added in a 1:1 ratio, resulting in a reduction in the overall peak for the gold core. XRD further confirmed the presence of a FCC crystal structure, with Bragg reflections corresponding well to those of Au–Pd BNPs, indicating a core-shell formation [50]. The calculated lattice parameters and crystallite size aligned well with the literature for similar noble metal core–shell systems. FTIR revealed successful binding of CS, 5-FU, DOX and Tf onto the surface of the BNPs as indicated by the presence of characteristic bands, corresponding to CS, 5-FU, DOX and Tf. The downshift of the aspartic acid-associated carboxyl peak in Tf, along with changes in the DOX and 5-FU bands, suggested coordination and encapsulation within the CS layer, as well as interaction with the BNP surface. These results correlated well with the literature [41,44,45,46,47,48,49].

TEM analysis highlighted the presence of spherical NPs with a dense Au core surrounded by a less dense Pd shell. The measured d-spacing values for the core and shell closely matched those expected for the (111) planes of Au and the (200) planes of Pd. These results confirmed that BNPs of a core–shell conformation were successfully synthesised [75]. NTA enabled the NP’s size and zeta potential to be accurately determined, with the zeta potential providing a reasonable estimation of NP colloidal stability [52,63,75]. The use of the PDI is valuable for determining a sample’s heterogeneity based on the particle size [76]. Studies have shown that NPs, which are monodisperse with a PDI < 0.3, serve better as stable and efficient delivery vehicles [77,78]. BNPs were smaller than 200 nm with a narrow size distribution (PDI < 0.2) and a high zeta potential, indicating good colloidal stability. Hence, these BNPs, along with their nanocomplexes, possessed the desirable sizes and zeta potentials for therapeutic delivery. Stability studies further confirmed these results, with the BNPs maintaining their size over several months.

A high (>70%) drug encapsulation was noted and was similar to those reported in previous studies [79,80]. It has been noted that the release of drugs from a polymer-based delivery system is influenced by the pH level of the microenvironment and any interactions between the respective drugs, i.e., 5-FU and DOX, and the CS layer [81]. It is further documented that at acidic pH, the amino groups present in CS ionize, resulting in swelling of the CS layer [82,83]. This causes the release of the encapsulated drugs through diffusion into the medium [84]. This allows for the selective release of the therapeutic payload into cancer cells, which have a more acidic microenvironment [85]. The release of both 5-FU and DOX from the CS-Tf-BMNPs was significantly higher at pH 4.2 and 5.2 when compared to pH 7.4. These results were consistent for both the single-drug and dual-drug BNP formulations and correlate with the literature. At acidic pH, the CS polymer chains undergo relaxation and protonation, resulting in an increase in the CS matrix pore size, which enhances swelling and accelerates drug diffusion [21,86,87]. This enabled the diffusion of the encapsulated drugs into the surrounding medium. In the dual-drug-CS-Tf-BMNPs, both swelling and diffusion were observed to contribute to the release of the drugs, particularly at lower pH levels. The higher drug release observed at pH 4.2 and 5.2 aligns well with the acidic microtumour environment, thereby supporting the selective release of the therapeutic payload at the tumour site.

Release kinetics enable researchers to optimise drug formulations and elucidate in vitro release mechanisms associated with NP delivery systems [88]. An effective model can help predict the behaviour of similar systems and explain the most likely mechanisms through which drug release occurs. These models can also help reduce experimental costs by highlighting limitations in NP design, allowing for optimisation of the formulation’s dose and release period, which is essential in determining the dose frequency for in vitro and in vivo studies [89]. A NP’s release kinetics depends primarily on the route of drug administration and the physicochemical properties of the NP [90,91]. These properties include NP size, surface coating and charge. Factors such as chemical composition, surrounding polymer matrix, concentration gradients, pH, fluctuations in the release environment, and the interactions between NPs, solvents and drugs influence an NP’s release profile [92,93]. The kinetic models employed in this study are commonly used in assessing the release of drugs from a delivery system. The drug release and kinetics studies demonstrated that the BNP nanocomplexes exhibited pH-responsive characteristics. The primary method of drug release occurred through diffusion.

The MTT cytotoxicity assay relies on the premise that viable cells possess higher mitochondrial activity, resulting in increased reduction in tetrazolium to formazan [94]. Cytotoxicity studies revealed that the BNPs exhibited negligible cytotoxicity in HEK293, HeLa, and MCF-7 cells, with an overall trend of improving cell viability with increasing concentration. These results align with the literature, which reports that noble metal NPs, including Au and Pd, are generally non-toxic due to their bioinert core [63,95]. This is important in ensuring that any observed cytotoxicity in the cells is not attributed to the BNPs, but rather to the functionalized drug contained within the nanocomplex. In the HEK293 cells, free 5-FU and DOX yielded the lowest cell viability. Upon encapsulation of the respective drugs with CS and conjugation to the BNPs with transferrin, there was an increase in cell viability, suggesting that the nanocomplexes reduced the cytotoxicity of 5-FU and DOX in normal cells. This most likely occurred through selective release and altered uptake mechanisms.

In contrast, the cancer cells (HeLa and MCF-7) exhibited a dose or concentration-dependent reduction in cell viability following treatment with the drug-loaded nanocomplexes. The highest levels of cytotoxicity were observed for the Tf-targeted formulations. In HeLa cells, the Tf-containing nanocomplexes (5-FU-CS-Tf-BMNPs, DOX-CS-Tf-BMNPs and dual drug-CS-Tf-BMNPs) produced the lowest cell viabilities. This could be due to the high expression of TfR on the surface of HeLa cells [37,96,97]. This resulted in enhanced cellular uptake by TfR-mediated endocytosis. Although MCF-7 cells also responded to Tf-targeted nanocomplexes, the effects were less apparent, which is consistent with lower Tf receptor expression in these cells. Overall, these findings correlate well with previous studies on Tf-tagged nanocarriers [37,87,98].

The receptor binding competition assay further confirmed evidence for TfR-mediated uptake, with a significantly reduced cytotoxicity of the Tf-targeted nanocomplexes upon pre-incubation with free Tf. Recent receptor competition studies using Tf and other ligands have shown a similar trend [87,98,99]. Overall, the cytotoxicity and receptor competition data confirmed that the CS-Tf-BNP nanocomplex exhibits selective cytotoxicity towards cancer cells, particularly the HeLa cells, which overexpress the Tf receptor. This is crucial in improving the therapeutic efficacy of 5-FU and DOX in cancer cells. These Tf-based drug nanocomplexes portend to be effective formulations with favourable preliminary results.

The combination of increased levels of reactive oxygen species (ROS), maintenance of redox homeostasis and increased antioxidant activity has contributed to cancer progression and metastasis [100,101,102]. ROS present a unique opportunity in cancer treatment, as they activate pro-apoptotic pathways, thereby aiding in the eradication of cancer cells [103]. Studies have demonstrated that ROS is essential to cellular apoptosis [104,105]. The oxidative stress and apoptosis studies provided additional confirmation of the mechanisms underlying the observed cytotoxicity of the nanocomplexes. The oxidative stress analysis revealed that BNP treatment resulted in a high percentage of ROS-negative cells in HEK293 cells, indicating that the BNPs were able to maintain or reduce oxidative stress in normal cells. These results correlate well with the low cytotoxicity of the BNPs in the HEK293 cells. The HeLa cells displayed resistance to oxidative stress, consistent with the literature [106,107], likely due to their enhanced redox buffering capacity, which is characteristic of cervical cancer cells. MCF-7 cells exhibited higher levels of ROS-positive cells, indicating that oxidative stress was a contributing factor to the observed cytotoxicity. Hence, these BNP nanocomplexes can exert their anticancer effects through a combination of cell specificity, targeted drug delivery and drug release in addition to their effect on redox homeostasis.

The AO/EB apoptosis studies highlighted that cell death in HeLa and MCF-7 cells was primarily apoptotic rather than necrotic. Acridine orange intercalates with cellular DNA, emitting a green fluorescence indicating living cells. In contrast, ethidium bromide stains dead cells and produces a yellow-to-red fluorescence [108,109]. The intensity and exact color of the fluorescence depend on the apoptotic stage of the cell. Although viable or healthy cells produce a green fluorescence, cells undergoing early apoptosis fluoresce a much brighter green, while late apoptotic cells fluoresce orange with condensed chromatin visible. Cells undergoing necrosis fluoresce a deeper orange to red, but with no condensed chromatin visible [72,110]. The overall results are consistent with the known mechanisms of 5-FU and DOX, which induce DNA damage, mitochondrial dysfunction and ROS generation, leading to apoptosis.

## 4. Materials and Methods

### 4.1. Materials

Palladium (II) chloride (Mw: 177.33 g·mol^−1^, PdCl_2_), gold (III) chloride trihydrate (Mw: 393.83 g·mol^−1^, HAuCl_4_·3H_2_O), chitosan ≥ 75% deacetylated (Mw: 1000–5000), sodium tripolyphosphate (Mw: 367.86 g·mol^−1^), Tween 80 (Mw: 1310 g·mol^−1^), Doxorubicin hydrochloride (Mw: 579.98 g·mol^−1^), 5-fluorouracil (Mw: 130.1 g·mol^−1^), acridine orange hemi (zinc chloride) salt (Mw: 265.36, g·mol^−1^), and dialysis tubing (MWCO = 1000 Daltons) were purchased from Sigma-Aldrich Chemical Co. (St. Louis, MO, USA). Phosphate-buffered saline tablets (PBS), sodium citrate tribasic, (3-(4,5-dimethylthiazol-2-yl)-2,5-diphenyl tetrazolium bromide (MTT), sodium borohydride (Mw: 37.83 g·mol^−1^), ethidium bromide, and dimethyl sulfoxide (DMSO), were purchased from Merck (Darmstadt, Germany). Human cell lines, including embryonic kidney (HEK293), cervical adenocarcinoma (HeLa), and breast adenocarcinoma (MCF-7), were originally obtained from the American Type Culture Collection (ATCC), Manassas, VA, USA, and were screened for mycoplasma prior to use in these studies. Eagle’s Minimum Essential Medium (EMEM) with L-glutamine (4.5 g·mL^−1^), penicillin/streptomycin/amphotericin B (100×) antibiotic mixture (25 mg·mL^−1^ of amphotericin B, 8.5 mg·L^−1^, 10,000 Units·mL^−1^ of penicillin, 10,000 mg·mL^−1^ of streptomycin sulphate), and trypsin-versene (versene (200 mg·L^−1^), trypsin (170,000 U·L^−1^)) were purchased from Lonza Bio Whittaker (Verviers, Belgium). Sterile fetal bovine serum (FBS) was obtained from Hyclone GE Healthcare (South Logan, UT, USA). Sterile tissue culture plasticware was obtained from Corning Inc. (New York, NY, USA), and 18 MOhm water (Milli-Q Academic, Millipore, Molsheim, France) was used in all reagents.

### 4.2. Methods

#### 4.2.1. Synthesis of Dihydrogentetrachloro-Palladate (H_2_PdCl_4_)

To synthesise H_2_PdCl_4_, 12 mL of HCl (0.2 M) and 500 mL of 18 M Ω water were added to a round-bottom boiling flask, followed by adding 0.1780 g of PdCl_2_. The pale-yellow solution was gently stirred for 20 min, refluxed for 3 h and then cooled. The resulting 2 mM H_2_PdCl_4_ solution was stored in a dark bottle and aged for 2 days before storage at 4 °C. 

#### 4.2.2. Synthesis of Gold Nanoparticles (AuNPs) and the AuNP-Core

An adapted Turkevich method was used to synthesise the AuNPs and the AuNPs core [111] and has been previously described by the authors [87].

#### 4.2.3. Synthesis of Gold-Palladium (AuPd) Bimetallic Nanoparticles (BNPs)

The shell structure for the BNPs was synthesised via an adapted protocol [112,113]. Briefly, 20 mL of a 1 mM AuNP (synthesised in Section 4.2.2) was gently stirred and refluxed. Thereafter, 20 mL of the 1 mM H_2_PdCl_4_ solution (synthesised in Section 4.2.1) was added dropwise over 12 min. The solution was refluxed for 40 min, with a colour change from ruby red to dark brown observed as the reaction progressed. The solution was cooled to room temperature, placed in a dark bottle and stored at 4 °C to minimise oxidation and photochemically induced structural changes. To ensure that the synthesized NPs displayed similar size, shape and physicochemical characteristics between batches, the protocol was optimized with regard to time, rate of addition of the H_2_PdCl_4_ solution, temperature, and rate at which the solution was stirred. The synthesised NPs demonstrated similar hydrodynamic sizes and zeta potentials, which were determined via NTA analysis.

#### 4.2.4. Synthesis of 5-FU and DOX-CS

For 5-FU/DOX encapsulation, 3.125 mL of 5-FU (3.8 mM) or 2.5 mL of DOX (1 mM) was added to 12.5 mL of CS (0.75 mg/mL in 2% acetic acid) with stirring. Tween 80 (0.5% *v*/*v*) was introduced into each drug solution and stirred for 15 min at a pH between 4.6–4.8. Thereafter, 6.25 mL of sodium tripolyphosphate (TPP, 1.4 mM) was added to each solution to achieve a CS:TPP ratio of 2:1 (*v*/*v*). The respective suspensions were stirred for 24 h to allow for complete drug (5-FU and DOX) encapsulation into the CS. The 5-FU-CS and DOX-CS solutions were stored at 4 °C.

#### 4.2.5. Binding of BNP to 5-FU-CS and DOX-CS

Approximately 4 mL of 5-FU-CS and DOX-CS solutions were added to separate conical flasks and gently stirred. The BNPs suspensions (2 mL) were added dropwise with stirring over 2 h. The 5-FU-CS: BNPs and DOX-CS: BNP ratio was maintained at (2:1 *v*/*v*).

#### 4.2.6. Formation of Dual Drug-CS-BNP

Briefly, 2 mL of DOX-CS and 2 mL of the 5-FU-CS were combined and stirred for 10 min. Thereafter, 2 mL of the BNP solution was added slowly over 2 h to enable efficient binding of the BNPs to the drug-CS complex. The dual drug nanocomplex formulation was then stirred in the dark for 5 h and subsequently stored at 4 °C.

#### 4.2.7. Synthesis of Transferrin (Tf) Targeted BNP Nanocomplexes

The concentration of Tf was maintained at 0.025 mg/mL for all nanocomplexes, i.e., 5-FU-CS-BNP, DOX-CS-BNP and dual drug-CS-BNP. Approximately 4 mL of 5-FU-, DOX- and dual drug-CS-BNPs from Section 4.2.5 and Section 4.2.6 were added to individual conical flasks with gentle stirring. This was followed by adding Tf (200 µL, 0.5 mg/mL) directly to the vortex of the solution. This was gently stirred for 12 h in the dark, and the final Tf-targeted BNPs were placed in a dark bottle and stored at 4 °C. Drug loading and transferrin attachment occurred through electrostatic interactions between the citrate-stabilised Au–Pd NP surface and the cationic CS-encapsulated drug formulations, as well as the functional groups of Tf. Electrostatic attraction between negatively charged regions on the NP surface and the positively charged amino groups of CS, together with hydrogen bonding and secondary coordination interactions, generated a stable bond without the need for carbodiimide coupling agents.

#### 4.2.8. UV-Visible (UV-Vis) Spectroscopy

All BNPs and nanocomplexes were analysed using UV-vis spectroscopy (Jasco V-730 UV-visible spectrophotometer, JASCO, Hachioji, Japan) to confirm their successful synthesis and conjugation. All samples were vortexed before analysis to ensure uniformity of the solution. Samples were analysed from 400 nm to 700 nm.

#### 4.2.9. Fourier Transform Infrared (FTIR) Spectroscopy

FTIR spectroscopy confirmed the presence of the essential functional groups and bonds in the respective nanocomplexes. All samples were analysed using a PerkinElmer Fourier-transform infrared spectrophotometer (Waltham, MA, USA) with a resolution of 4 cm^−1^, and a wavelength range from 4000 cm^−1^ to 650 cm^−1^. The IR spectra were generated using the built-in Spectrum Analysis Software 10.

#### 4.2.10. Inductively Coupled Plasma-Optical Emission Spectroscopy (ICP-OES)

Inductively coupled plasma-optical emission spectroscopy (ICP-OES) was carried out on a Perkin Elmer Optima 5300 DV optical emission spectrometer (Waltham, MA, USA). A standard curve was established from 1 and 20 ppm using gold and palladium standards at a concentration of 100 ppm. ICP-OES enabled elemental detection and quantification and was used to determine the concentration of gold and palladium in the respective colloidal NP solutions.

#### 4.2.11. X-Ray Diffraction Analysis (XRD)

XRD analysis enabled the investigation of crystallinity and phase purity of the NP formulations. All XRD patterns were recorded using a Malvern Pananalytical Aeris diffractometer (Malvern, Worcestershire, UK) with a PIXcel detector and fixed slits with Fe-filtered Co-Kα radiation. The phases were identified using X-Pert Highscore Plus V 5.3a software. The XRD spectra were recorded in the 2θ range of 10° to 80°.

#### 4.2.12. Transmission Electron Microscopy (TEM)

TEM is a powerful tool which enables NP size and morphology to be determined. The BNPs and nanocomplex solutions (10 µL) were added to a carbon-coated copper grid (400-mesh) (Ted Pella Inc., Redding, CA, USA) and air-dried at room temperature for 1 h. Samples were viewed at 60,000× magnification using a JEOL-JEM T1010 (Jeol, Tokyo, Japan) electron microscope without warming above −150 °C at an acceleration voltage of 100 kV. The iTEM Soft Imaging Systems (SIS) Megaview III fitted with a side-mounted 3-megapixel digital camera (Jeol, Tokyo, Japan) was used to capture the TEM images.

#### 4.2.13. Nanoparticle Tracking Analysis (NTA)

NTA determined the hydrodynamic sizes and zeta potentials (ZP) of the BNPs and nanocomplexes. NTA was conducted in a Nanosight NS-500 (Malvern, Worcestershire, UK) operating at 25 °C. Samples (1 mL) were diluted 1:500 (in 18 M Ω water) before analysis. Data are presented as mode ± standard error.

#### 4.2.14. Drug Encapsulation Efficiency

The drug encapsulation efficiency (EE) was assessed by centrifuging 500 µL of the respective drug-encapsulated nanocomplexes at 15,000 rpm for 1 h at 15 °C. Absorbance readings determined for 5-FU (at 266 nm) and DOX (at 481 nm) were used to calculate the total drug present. Equation (1) was used to determine the drug EE.

(1) EE (%)=Total Drug−Free Drug/Total Drug ×100

#### 4.2.15. Drug Release Profiles and Kinetic Studies

Drug release studies were conducted over 72 h at pH levels of 4.2, 5.2, and 7.4. Approximately 300 µL of each nanocomplex was dialysed (MWCO = 1000 Da) against 6 mL PBS for 72 h at 37 °C with stirring. For analysis, 10 µL of respective samples were removed at fixed intervals and analysed on a Jasco V-730 UV-visible spectrophotometer (JASCO, Hachioji, Japan) at 266 nm (5-FU) and 481 nm (DOX), respectively. These UV-vis results generated a cumulative drug release profile using Equation (2).

(2) $$\mathrm{Cumulative}\;\mathrm{Drug}\;\mathrm{Release}\;(\%)=\frac{Total\;Released\;Drug}{Total\;Encapsulated\;Drug}\times 100$$

Various mathematical-based models have been developed to determine the mechanisms of drug release. The models used in this study were the zero-order [114,115], first-order [116,117], Higuchi [118], Hixon-Crowell [119], and Korsmeyer-Peppas [120] models. The Kopcha model is essential in determining the mechanism through which a drug is released from a NP in relation to drug diffusion and polymer relaxation [121,122]. The correlation coefficient, expressed as (R2), enables the most suitable release model to be selected. The correlation coefficient and the release exponents described the release mechanism. The models and Equations (3)–(8) are listed below:

The Kopcha model [121,122]:

(3) $$Q_{t}=A_{t}\;\frac{1}{2}+B_{t}$$

where t is time, A is the diffusion constant, B is the erosion constant, and Q_t_ is the amount of drug dissolved in time t.

The Zero-order kinetic model [114,115]:

Qt = Q_0_ + K_0_t (4)

where Q_t_ is the quantity of drug released in time (t), Q_0_ is the initial amount of drug present (usually Q_0_ = 0), K_0_ is the zero-order release constant (concentration/time), and t is time.

First-order kinetic model [116,117]:

(5) $$\log Q_{t}=\log Q_{0}+\frac{k_{1}t}{2.303}$$

where Qt is the quantity of drug released in time (t), Q0 is the initial quantity of drug (usually Q0 = 0), K1 is the first-order release constant (concentration/time), and t is time.

Hixon-Crowell kinetic model [119]:

(6) $$W_{0}\;\frac{1}{3}\;-\;W_{t}\;\frac{1}{3}={K_{HC}}^{t}$$

where W_t_ is the quantity of drug released after time (t), W_0_ is the initial amount of drug present, and K_HC_^t^ is the rate constant for the Hixon-Crowell rate equation.

The Higuchi kinetic model [118]:

(7) $$Q_{t}=Q_{0}+K_{H}\;t_{2}^{1}$$

where Qt is the amount of drug released in time t, Q_0_ is the initial amount of drug in the solution, and KH is the Higuchi constant.

The Korsmeyer-Peppas kinetic model [120,123]:

F = M_t_/M_n_ = K_kp_·t^n^ (8)

where *M_t_*/*M_n_* is an expression of the fraction of drug released at time (t), *K_kp_* is the Korsmeyer-Peppa’s rate constant. which is affected by the structure and the geometric properties of the system, and *n* is the release exponent. Peppa used *n* to describe release through alternative mechanisms [123]. When *n* = 0.43, the drug release occurs through Fickian diffusion, which does not result from any relevant deformation or stresses. When *n* < 0.43, the release mechanism is considered as Quasi-Fickian diffusion. When *n* is between 0.43 < *n* < 0.85, the release is associated with anomalous diffusion, which can result from structural stress or swelling. If *n* > 0.85, case II transport occurs, and when *n* = 1, Zero-order release kinetics can be observed [124]. Mathematical models that generate correlation coefficients greater than 75% are considered suitable. The correlation coefficient is a good determinant of which model best fits the release data

#### 4.2.16. In Vitro Cytotoxicity—MTT Assay

Cells were seeded at a density of 1.5 × 10^4^ cells per well into a 96-well plate, and incubated at 37 °C in 5% CO_2_. Thereafter, the cells were treated with the BNPs and nanocomplexes at the specified ratios in Table 10 and then incubated for an additional 48 h. The growth medium was then replenished with 100 µL fresh medium containing 10 µL of MTT reagent (5 mg/mL in PBS), and cells were incubated for 4 h at 37 °C. The MTT-media was removed, and 200 µL of DMSO was used to solubilise the formazan crystals. Absorbance was read on a Mindray MR-96A microplate reader (Vacutec, Hamburg, Germany) at a wavelength of 570 nm, using DMSO as a blank. Positive controls (cells only) were set as 100% cell viability. Cell viability was calculated using Equation (9).

(9) $$\mathrm{Cell}\;\mathrm{Viability}\;(\%)=\frac{\left(Absorbance\;of\;Treated\right)-(Absorbance\;of\;control)}{\left(Absorbance\;of\;control\right)}\times 100$$

#### 4.2.17. Receptor Binding Competition Assay

This assay was used to determine if the cellular uptake mechanism was via Tf-mediated endocytosis. Cells were prepared and incubated as in Section 4.2.16. The spent medium was decanted, and fresh medium (100 µL) was added. TF (200 µM), in excess of that which was contained in the nanocomplexes, was added, and cells were incubated for 20 min. The 5-FU-CS-Tf-BNP, DOX-CS-Tf-BNP, and dual drug-CS-Tf-BNP nanocomplexes at the highest treatment ratio (Table 11) were added to the cells, which were then incubated at 37 °C for 48 h and analysed as described in Section 4.2.16.

#### 4.2.18. Intracellular Oxidative Stress Analysis

Quantitative measurement of reactive oxygen species was obtained on a MUSE^™^ cell analyzer (Luminex, Austin, TX, USA) using the Muse^®^ Oxidative stress kit. Cells were subcultured at a density of 1 × 10^5^ cells/mL into 96-well plates and incubated overnight at 37 °C. After 24 h, the spent medium was removed, and 100 µL of fresh medium (EMEM + 10% FBS + 1% antibiotics) was added, followed by the addition of individual nanoparticles and complexes at the concentrations specified in Table 11. Cells were incubated for 48 h at 37 °C in 5% CO_2_. Following a 48 h incubation, cells were pelleted at 300× *g* for 5 min and then washed with PBS. Cells were prepared in 1× assay buffer for incubation with Muse^®^ oxidative stress working solution. For incubation, 190 µL of Muse^®^ oxidative stress working solution was added to 10 µL of cells and incubated at 37 °C for 30 min in the dark. The ROS^−^ and ROS^+^ cell populations were measured by using a Muse^®^ cell analyser and its in-built software (Luminex Corporation, Austin, TX, USA).

#### 4.2.19. Apoptosis Assay

This is a fluorescence-based assay to detect apoptosis in cell culture. The assay utilises a dual dye consisting of AO and EB. Initially, each dye was prepared at 100 mg/mL (in PBS) and used in a 1:1 (*v*/*v*) ratio. Cells at a density of 1.2 × 10^5^ cells per well were seeded into 24-well plates and incubated at 37 °C overnight. The spent medium was replenished with fresh medium (500 µL), and the different NPs and nanocomplexes in triplicate were added at concentrations corresponding to their calculated IC_50_ values (Table 12). Positive controls contained untreated cells. Cells were incubated for 48 h at 37 °C in 5% CO_2_ and washed with PBS (100 µL). The dye solution (10 µL) was then added to the cells. Cells were viewed under an Olympus fluorescence microscope (200× magnification) fitted with a CC12 fluorescent camera (Olympus Co., Tokyo, Japan). The apoptotic indices were calculated using Equation (10).

Apoptotic index = Number of Apoptotic cells/total number of cells(10)

#### 4.2.20. Statistical Analysis

All data from this study are presented as mean ± standard deviation (±SD *n* = 3). Assays were conducted in triplicate unless stated otherwise. Dunnett’s post hoc test was used for the cytotoxicity assays. The statistical significance of the tests was set at ** *p* < 0.01 and * *p* < 0.05. Each experimental value was compared to its corresponding control. The software used for statistical analysis was GraphPad Instat 3 (GraphPad Software, Inc., San Diego, CA, USA). Microsoft Excel 365™ (Microsoft, Redmond, WA, USA) and Excel Add-in DD solver and Data Analysis (Microsoft, Redmond, WA, USA) software were employed to evaluate the release kinetics parameters.

## 5. Conclusions

This study successfully synthesised the BNPs and their functionalisation with the cationic polymer chitosan containing 5-FU and DOX, and conjugated to the targeting ligand, transferrin. The BNPs displayed favourable properties and anticancer activities, which could be attributed to the synergism of the two metals in the NP formulation. Importantly, this study confirmed the Tf-receptor-mediated uptake of the nanocomplexes in the HeLa cells, boding well for their use in cervical cancer therapy. Overall, the targeted BNPS enhanced the cytotoxicity in cancer cells (HeLa and MCF-7) while improving cell viability in non-cancer cells (HEK293). BNPs also generated a more favourable and controlled drug delivery release profile, desirable for therapeutic applications. Overall, the BNPs have shown the potential to be efficient drug delivery nanocarriers, warranting further investigation. In addition, optimisation regarding the amount of targeting ligand and chitosan used can be undertaken, along with further mechanistic studies to elucidate the mechanisms involved in cell death. The success of transferrin-targeting can be further explored by examining transport across the blood-brain barrier, which can be facilitated by transferrin. Lastly, further investigation in an in vivo model is warranted. The findings of this study provide strong preliminary evidence that Tf-targeted, CS-coated Au-Pd BNPs represent a promising nanoplatform for the controlled, pH-responsive and targeted delivery of 5-FU and DOX. This system has the potential to improve the efficacy and safety of established chemotherapeutic agents and can be adapted to other drug combinations in future studies. While the in vitro findings provide strong evidence for the targeted delivery and cytotoxic efficacy of the BNP nanocomplexes in HeLa cells, they do not fully account for the complexity of the tumor microenvironment or the systemic interactions that direct NP behaviour in vivo. Physiological processes such as serum protein adsorption, immune recognition, vascular permeability, interstitial transport, and NP clearance cannot be adequately reproduced in culture systems and may substantially influence NP distribution, stability, and cellular uptake in vivo [125,126].

Further investigations will also examine long-term biocompatibility, dose-dependent toxicity, and clearance pathways, as metal-based NPs may undergo surface modification through protein corona formation, oxidative reactions, or partial dissolution in biological fluids. The synthesis protocol developed for the BNPs involved mild reaction conditions and readily available reagents, thereby indicating its potential suitability for scale-up processes. However, large-scale production would necessitate further optimization to ensure batch uniformity, efficient purification, and improved reproducibility [127]. The long-term biocompatibility needs to be assessed, as noble metal NPs may undergo surface modification or interact with biomolecules over extended periods. Comprehensive evaluations of immune activation and clearance pathways will therefore be required to support the future development of this nanosystem.

Future work will therefore focus on optimising synthesis scalability, refining ligand density to enhance tumour specificity, and evaluating therapeutic performance in relevant cervical cancer models. Additional investigations will also focus on evaluating the potential for combination therapy and conducting direct comparisons with monometallic counterparts. These studies will provide a more comprehensive understanding of the translational potential of these BNPs from the laboratory to the clinic.

## Figures and Tables

**Table 1:** Characteristic peaks for transferrin, chitosan, DOX, and 5-FU in the FTIR spectra.

Molecule	Characteristic Peak (cm^−1^)	Assignment
Chitosan (CS)	3000–3600 (broad)	O–H and N–H stretching
	1648	Amide I (C=O stretch)
Transferrin (Tf)	1635	Amide I/histidine stretching
	1402	Aspartic acid—COO^−^ symmetric stretch
5-FU (5-FU)	2800–3100	C–H (imide) stretching
	1431–1658	C=C and C=N stretching
	~1398	Aromatic ring vibration
Doxorubicin (DOX)	3382	O–H stretch
	1618	C=O/aromatic stretching

**Table 2:** Peak list for the AuPd BNPs.

No.	h	k	l	d [Å]	2θ [°]	I [%]
1	1	1	1	2.33249	45.101	100.0
2	0	0	2	2.02000	52.568	47.9
3	0	2	2	1.42836	77.548	28.8
4	1	1	3	1.21811	94.503	32.7
5	2	2	2	1.16625	100.169	9.4

**Table 3:** NTA-based size, zeta potential and polydispersity indices of the nanoparticles and their respective nanocomplexes.

Sample	Size (nm)	Zeta Potential (mV)	PDI (σ/D)^2^
AuNP	73.4 ± 9.8	−16.3 ± 2.3	0.101239669
5-FU-CS	130.3 ± 13.6	21.4 ± 0.2	0.16599859
DOX-CS	128.7 ± 3.5	18.3 ± 0.8	0.054837146
5-FU-CS-BNP	85.9 ± 8.4	16.1 ± 0.3	0.108083068
DOX-CS-BNP	96.5 ± 48	5.4 ± 6.5	0.164671288
Dual-Drug-CS-BNP	91.8 ± 47.2	2.8 ± 0.4	0.194212819
5-FU-CS-Tf-BNP	105.6 ± 10.7	16.9 ± 0.3	0.138109753
DOX-CS-Tf-BNP	107.1 ± 18.1	14.4 ± 1.8	0.098792686
Dual-Drug-CS-Tf-BNP	112.8 ± 0.9	9.4 ± 1.9	0.081632653

**Table 4:** NTA-based size distribution and zeta potential assessment of the BNPs.

Day	Bimetallic Nanoparticle Size (nm)	Zeta Potential (mV)
0	73.4 ± 9.8 nm	−28.7 ± 0.2 mV
7	52.1 ± 2.9 nm	−36.2 ± −7.0 mV
14	83.8 ± 21.9 nm	−31.5 ± 0.4 mV
21	94.0 ± 6.4 nm	−25.7 ± 0.4 mV
28	61.5 ± 4.3 nm	−27.3 ± 6.0 mV
216	76.01 ± 5.9 nm	−69.27 ± 2.0 mV

**Table 5:** Correlation coefficients (R2) obtained from 5-FU-CS-Tf-BNP through release kinetic models at pH 4.2, 5.2 and 7.4.

pH	Zero-Order	First-Order	Higuchi’s	Hixon-Crowell’s	Korsmeyer-Peppa’s	Kopcha’s
4.2	0.8756	0.9143	0.963	0.9029	0.5008 *n* = 0.52	0.9173
5.2	0.792	0.08674	0.9602	0.845	0.604 *n*= 0.43	0.9879
7.4	0.8201	0.873	0.9375	0.8569	0.6338 *n*= 0.56	0.9884

*n* = calculated Korsmeyer-Peppa’s release exponential.

**Table 6:** Diffusion (A) and erosion (B) constants obtained from 5-FU-CS-Tf-BNP through Kopcha’s release kinetic model at pH 4.2, 5.2 and 7.4.

pH	Kopcha Model		
	A	B	A/B
4.2	210.12	99.098	2.120325
5.2	16.132	0.266	60.64662
7.4	10.71	0.7688	13.9308

**Table 7:** Correlation coefficients (R2) obtained from DOX-CS-Tf-BNP through release kinetic models at pH 4.2, 5.2 and 7.4.

pH	Zero-Order	First-Order	Higuchi’s	Hixon-Crowell’s	Korsmeyer-Peppa’s	Kopcha’s
4.2	0.9522	0.9694	0.9559	0.9679	0.5972 *n* = 0.65	0.9223
5.2	0.8428	0.9087	0.9782	0.889	0.644 *n* = 0.43	0.9728
7.4	0.7659	0.8069	0.926	0.7938	0.6903 *n* = 0.30	0.9989

*n* = calculated Korsmeyer-Peppa’s release exponential.

**Table 8:** Diffusion (A) and erosion (B) constants obtained from DOX-CS-Tf-BNP using the Kopcha release kinetic model at pH levels of 4.2, 5.2, and 7.4.

pH	Kopcha Model		
	A	B	A/B
4.2	17.585	0.8439	20.83778
5.2	13.64	0.8158	16.71978
7.4	8.3373	0.5348	15.58957

**Table 9:** Correlation coefficients (R2) obtained from dual drug-CS-Tf-BNP through release kinetic models at pH 4.2, 5.2 and 7.4.

pH	Zero-Order	First-Order	Higuchi’s	Hixon-Crowell’s	Korsmeyer-Peppa’s	Kopcha’s
	5-FU/DOX	5-FU/DOX	5-FU/DOX	5-FU/DOX	5-FU/DOX	5-FU/DOX
4.2	0.8535/0.7818	0.9232/0.8575	0.9734/0.9604	0.902/0.902	0.7554; *n* = 0.58/ 0.4422; *n* = 0.77	0.923/0.9785
5.2	0.8089/0.7172	0.9022/0.8243	0.9739/0.8996	0.8741/0.79	0.5029; *n* = 0.73/ 0.7209; *n* = 0.31	0.2472/0.9826
7.4	0.695/0.7216	0.7453/0.7807	0.8758/0.9186	0.727/0.7618	0.6833; *n* = 0.56/ 0.9857; *n* = 1	0.9859/0.9822

*n* = calculated Korsmeyer-Peppa’s release exponential.

**Table 10:** Diffusion (A) and erosion (B) constants obtained from dual drug-CS-Tf-BNP through the Kopcha release kinetic model at pH 4.2, 5.2 and 7.4.

pH	Kopcha Model					
	A		B		A/B	
	5-FU	DOX	5-FU	DOX	5-FU	DOX
4.2	7.166	31.687	1.2963	1.4891	5.528041348	21.27929622
5.2	24.082	4.6556	2.0621	2.6393	11.67838611	1.763952563
7.4	9.1559	10.451	0.3707	0.5873	24.69894794	17.79499404

**Table 11:** The ratios of the BNPs and nanocomplexes utilized in the MTT assays.

Nanoparticle/Nanocomplex	Ratio	Nanocomplex Ratio Drug Conc		
		1	2	3
BNP	1:0	0.134 µg	0.268 µg	0.402 µg
Free DOX	1:0	0.1701 µg	0.3401 µg	0.5103 µg
Free 5-FU	1:0	0.1701 µg	0.3401 µg	0.5105 µg
5-FU-CS	1:4 = 1 *	0.1702 µg	0.3404 µg	0.5106 µg
DOX-CS	1:5 = 1 *	0.1701 µg	0.3402 µg	0.5103 µg
5-FU-CS-BNP	1:2 = 1 *	0.1701 µg	0.3403 µg	0.5105 µg
DOX-CS-BNP	1:2 = 1 *	0.1701 µg	0.3402 µg	0.5103 µg
Dual Drug-CS-BNP	1:1:1 = 1 *	0.1701 µg	0.3402 µg	0.5104 µg
5-FU-CS-Tf-BNP	20:1	0.1701 µg	0.3403 µg	0.5105 µg
DOX-CS-Tf-BNP	20:1	0.1701 µg	0.3402 µg	0.5103 µg
Dual Drug-CS-Tf-BNP	20:1	0.1701 µg	0.3402 µg	0.5104 µg

Where * denotes complex, taken as a single unit for comparison with the subsequent addition of ligand/nanoparticle.

**Table 12:** Nanoparticle/nanocomplex IC_50_ concentrations.

Nanoparticle/Nanocomplex	IC_50_ Concentrations (µg)		
	HEK293	HeLa	MCF-7
BNP	4.90	3.12	5.56
Free 5-FU	0.062	0.463	0.062
Free DOX	0.103	0.351	0.038
5-FU-CS	0.160	0.670	0.502
DOX-CS	0.133	0.244	0.136
5-FU-CS-BNP	1.286	0.968	1.997
DOX-CS-BNP	0.650	0.183	0.972
Dual Drug-CS-BNP	3.183	0.491	0.763
5-FU-CS-Tf-BNP	3.998	0.461	2.221
DOX-CS-Tf-BNP	3.741	0.221	1.324
Dual Drug-CS-Tf-BNP	3.104	0.0265	1.241

**Table 13:** Apoptotic indices for the BNP nanocomplexes in HEK293, HeLa and MCF-7 cells.

Sample	HEK293	HeLa	MCF-7
BNP	0.07	0.14	0.12
Free 5-FU	0.35	0.81	0.35
Free DOX	0.41	0.86	0.61
5-FU-CS	0.31	0.72	0.29
DOX-CS	0.38	0.71	0.49
5-FU-CS-BNP	0.29	0.77	0.47
DOX-CS-BNP	0.25	0.84	0.43
Dual Drug-CS-BNP	0.22	0.86	0.42
5-FU-CS-Tf-BNP	0.19	0.85	0.28
DOX-CS-Tf-BNP	0.22	0.89	0.31
Dual Drug-CS-Tf-BNP	0.21	0.96	0.34

## Data Availability

The original contributions presented in this study are included in the article. Further inquiries can be directed to the corresponding author.

## Abbreviations

The following abbreviations are used in this manuscript:

5-FU	5-Fluorouracil
DOX	Doxorubicin
AuNP’s	Gold nanoparticles
PdNP’s	Palladium nanoparticles
BNP’s	Bimetallic nanoparticles
CS	Chitosan
NTA	Nanoparticle tracking analysis
HEK293	Human embryonic kidney
HRTEM	High resolution transmission electron microscopy
Mw	Molecular weight
mM	Millimolar
g	Grams
DMSO	Dimethyl sulfoxide
FBS	Fetal bovine serum
gml	Grams per ml
M	Molar
ml	Millilitre
nm	Nanometre
SPR	Surface plasmon resonance
TEM	Transmission electron microscopy
FTIR	Fourier transform infra-red analysis
XRD	X-Ray diffraction analysis
ICP	Inductively coupled plasma
ul	Microlitre
mV	Millivolt
Tf	Transferrin
